# A Qualitative Analysis of Pediatricians’ Perspectives Regarding Barriers, Facilitators, and Strategies for Providing Timely Autism Spectrum Disorder Screening and Diagnosis

**DOI:** 10.1177/11795565261431178

**Published:** 2026-04-06

**Authors:** Mallika Mathur, Jinni Tang, Christine Markham, Sandra McKay, Ruosha Li, Deepali Ernest, Allison Marshall, Shreela Sharma

**Affiliations:** 1 Center for Healthy Communities, Department of Epidemiology, School of Public Health, The University of Texas Health Science Center at Houston, USA; 2 Department of Pediatrics, McGovern Medical School, The University of Texas Health Science Center at Houston, USA; 3 Department of Health Promotion and Behavioral Sciences, School of Public Health, The University of Texas Health Science Center at Houston, USA; 4 Department of Biostatistics and Data Science, School of Public Health, The University of Texas Health Science Center at Houston, USA; 5 Department of Human Services, Georgetown University School of Health, Washington, DC, USA

**Keywords:** pediatrician, children, autism, barriers, strategies, diagnosis

## Abstract

**Background:**

Autism spectrum disorder (ASD) is a developmental disorder that has increased in prevalence over the years. Diagnosis and subsequent intervention by the age of 3 is essential for development. However, recent estimates indicate that less than 50% of children receive an evaluation by age 3. Underserved populations are more likely to receive an evaluation at an older age. Several barriers in the diagnostic process have been identified; however, limited studies have aimed at understanding the specifics of these barriers among underserved populations and whether current strategies are beneficial for all children.

**Objectives:**

The aim of this study was to identify barriers and strategies, as reported by pediatricians serving underserved populations, to enhance timely screening and diagnosis of ASD.

**Design:**

A qualitative study was conducted.

**Methods:**

Semi-structured interviews were conducted with general pediatricians and subspecialists (N = 14) practicing in the Greater Houston area, Texas. Thematic analysis was conducted using inductive reasoning.

**Results:**

Participants were majority White (50%), female (79%), practicing in hospital-based settings (64%), with 3 to 5 years of professional experience (64%) and a mean age of 50 years. Three themes were identified (1) barriers causing delays in ASD screening and diagnosis; (2) individualized approaches in pediatric care to address barriers, and (3) recommendations to improve timely ASD screening and diagnosis.

**Conclusion:**

The results of this study identified potential strategies to overcome access, availability, and knowledge barriers; however, the lack of standardization across the health system results in the continued reporting of significant barriers. Using a health equity approach is key to ensure improvements in timely screening and diagnosing by tailoring strategies to the population being served. Health systems should work on expanding and evaluating identified strategies. Additionally, improve education for providers and parents to address parental hesitancy, increase attendance with routine well-child checks, and improve ASD evaluation referrals.

## Introduction

Autism Spectrum Disorder (ASD) is classified as a developmental disorder associated with deficits in social interactions, communication, and restricted or repetitive behaviors.^1,2^ In the United States (U.S.), the most recent estimates report that 1 in 31 children 8 years of age are diagnosed with ASD.^2^ The American Academy of Pediatrics (AAP) recommends that ASD-specific screening occur for all children between 18 and 24 months during routine well-child visits with a general pediatrician, using validated screeners.^3^ A positive screen typically results in a referral to a subspecialist (ie, developmental pediatrician, trained general pediatrician, and psychologists) for a developmental evaluation to receive an official ASD diagnosis.^3^ Receiving an early diagnosis is important for growth and development, as it allows for early initiation of key therapies such as speech therapy and applied behavioral analysis (ABA) therapy, which can greatly impact the short- and long-term cognitive and health outcomes of ASD.^4^ Despite these guidelines, the Autism and Developmental Disabilities Monitoring (ADDM) network reports that less than half of the children with ASD receive an ASD developmental evaluation for a diagnosis by the age of 3.^5^

Underserved populations, such as low-income families, rural communities, and minority groups, are more likely to report older ages of ASD diagnosis.^6-9^ Past qualitative studies conducted within the U.S. have highlighted several persistent barriers influencing timely access to ASD screening and diagnosing. Commonly reported barriers include a lack of parental knowledge, biases and low confidence of providers in addressing a positive ASD screen, and issues in healthcare access due to specialist shortages.^10-14^ Underserved populations encounter added barriers such as inadequate insurance coverage, financial constraints, limited health literacy, transportation difficulties, and geographic gaps in service.^15-17^ Strategies such as general provider training to evaluate ASD in primary care settings and improve access have been developed to overcome barriers.^18,19^ However, there is limited research aimed at understanding the nuances of these barriers among underserved populations and whether current strategies are feasible for all children. Understanding the specifics of the barriers faced by underserved populations is critical to reduce health disparities and ensure successful widespread incorporation of strategies into the health system.^20^ Moreover, the COVID-19 pandemic exacerbated access issues, particularly among underserved populations, resulting in fewer children receiving routine checkups,^21^ therefore delaying when a child received an ASD screening and diagnosis. In response to COVID-19, telehealth was used more widespread to improve access to screening and diagnosing services.^22^ However, limited studies have examined whether telehealth was beneficial, especially for children from low-income households or rural communities. More detailed insight is needed from pediatricians, who are responsible for assessing children for ASD, to determine whether alternative strategies should be incorporated into the current screening and diagnosing practices and whether the pandemic highlighted additional barriers preventing children from receiving a timely ASD diagnosis.^11^

The observed disparities in age of diagnosis warrant the need for further evidence aimed at understanding the barriers and facilitators among underserved populations.^6-9^ To address this need, we designed a qualitative research study to help guide the development of practical approaches to address delays in ASD screening and diagnosis.^11^ Therefore, the purpose of our study was to explore barriers and strategies, as identified by pediatricians serving underserved populations, to improve timely ASD screening and diagnosis for all children.

## Methods

### Participants

Purposive sampling was used to recruit general pediatricians and subspecialists (developmental pediatricians and pediatric neurologists) from the greater Houston area, Texas. Participants were included in the study if they were (1) a healthcare professional who is qualified to provide an ASD screening and/or diagnosis, (2) had been practicing for at least 2 years, ensuring that they had sufficient experience to provide insights, and (3) were working in practices or clinics where at least 15% of patients served were Medicaid recipients, so that perspectives related to patients from all socioeconomic backgrounds would be included. Participants were excluded if their practices or clinics did not accept Medicaid. Interviews were conducted until data saturation was reached among both general pediatricians and subspecialists, meaning no new themes or insights emerged, resulting in a total sample of 14 participants.^23^ Although the subspecialist sample was smaller, saturation was achieved as interviews yielded consistent themes. Overarching themes were identified across both groups, while differences in clinical roles, visit types, and patient populations were also considered to ensure each group’s perspectives were adequately represented. Data saturation was evaluated by 2 researchers trained in qualitative methods, MM and JT, and was reached after 11 interviews. An additional 3 interviews were conducted after a consensus of data saturation was reached to ensure all relevant information was captured.

### Procedure

Pediatrician interviews were conducted using a semi-structured interview guide (Table 1). The institutional review board of the University of Texas Health Science Center at Houston approved the study (HSC-SPH-23-0508). Since this study was considered minimal risk and did not collect sensitive participant information, informed verbal consent was obtained from participants prior to engaging in the interview and after thorough review of the letter of information.

**Table 1.** Interview Guide.

Topic	Questions
Awareness of practices related to ASD screening, referral and diagnosing	1. Briefly, at your clinic, how do you screen and/or diagnose for ASD? (Probes: What are the criteria involved? Who does it? At what age does the child typically get screened?) 2. What are your referral practices for a child that screens positive? 3. Are there benefits for early ASD screening and diagnosing for children and their families? If so, what?
Barriers and facilitators	4. What are the facilitators to an early screening and/or diagnosis? (eg, Are there factors or clinic processes that make it easier for you to conduct early screenings of your patients?) 5. What are the barriers to early screening and/or diagnosis? 6. What are the facilitators and barriers, if any, for placing a referral for every child screening positive? 7. Are any barriers preventing certain populations from receiving an earlier screening or diagnosis?
COVID-19 related impacts and strategies	8. What impact did the COVID-19 pandemic have, if any, on ASD screening and diagnosing? 9. Did you make any changes in regards to ASD screening, diagnosing and referral practices due to the pandemic? a. If yes, how useful were the changes you made? b. Do you think these changes will be helpful in addressing the barriers you mentioned earlier?
Future improvements	10. What would you like to see changed or improved that would make screening, diagnosis, and referral processes easier or smoother or more effective? 11. Are there any other comments you would like to make on ASD screening, diagnosis and referrals that would help me understand the challenges and opportunities better?

### Study Recruitment and Data Collection

Using a list of pediatricians in the area obtained from the medical school, participants were recruited via email between August 2023 and October 2023 with a flyer detailing the study parameters. Interested potential participants reached out to MM and SM, and then were scheduled for interviews by MM. The interview sessions were conducted by author MM between August 2023 and November 2023. These interviews were conducted virtually using Microsoft Teams, a video conferencing platform, to accommodate the fluctuating and complex schedules of practicing physicians. After agreeing to participate in the study, participants were asked to complete a brief demographic survey before the virtual interviews. The demographic survey collected the participants’ age, years of experience as a pediatrician, race and ethnicity, sex, and clinic location. Practice type, hospital-based or private clinic, was collected through participants’ job profiles (Table 2). Participants provided consent for all interviews to be audio-recorded and transcribed.

**Table 2.** Participants’ Characteristics (N = 14).

Participant characteristics (N = 14)	N (%)
Mean age (SD)	49.6 (12.2)
Sex	
Male	3 (21.4%)
Female	11 (78.6%)
Race/ethnicity	
White, Caucasian, or Anglo	7 (50.0%)
Asian (Chinese, Indian, or another Asian Country)	6 (42.9%)
Mexican American, Latino or Hispanic	1 (7.1%)
Practice type	
Hospital based	9 (64.3%)
Private	5 (35.7%)
Years of experience	
2 y	1 (7.1%)
3-5 y	9 (64.3%)
6-10 y	3 (21.4%)
10+ y	1 (7.1%)

A semi-structured interview guide was developed after a detailed review of the literature and guidance from experts including practicing pediatricians and qualitative researchers. The interview guide included topics related to the pediatrician’s awareness of practices related to ASD screening and diagnosing, barriers to screening and diagnosing faced by children and their families, challenges faced by healthcare providers, impacts of the COVID-19 pandemic on ASD screening/diagnosis, strategies used to overcome these barriers, and the effectiveness of the strategies. After completing 5 interviews, the interview guide was reassessed by the researchers to ensure the questions were clear and no additional questions were required. No significant changes were made to the guide upon review.

### Data Analysis

Descriptive statistics were conducted to examine means, standard deviations, frequencies, and percentages of the participants’ demographic characteristics. The interviews were analyzed using reflexive thematic analysis, a qualitative approach for identifying and interpreting themes across data.^24^ Reflexive thematic analysis emphasizes the researcher’s active role in generating themes through iterative coding, interpretation, and reflexive engagement with the data.^24^ We selected reflexive thematic analysis because it offers a flexible yet rigorous way to identify themes while acknowledging the researcher’s active role in shaping them. Recordings of the interviews were transcribed by author MM. Thematic analysis using inductive reasoning was conducted by 2 trained qualitative researchers, MM and JT. ATLAS.ti Web (version 5.8.0)^25^ was used to systematically apply the codes that emerged from words and concepts grouped together.^26^ The 2 researchers both analyzed the data separately to ensure the validity and comparability of the results. After each transcript was reviewed, initial codes were generated, and the data were organized at a granular level. Atlas.ti Web was used to organize codes, compare coding across researchers, and track analytic decisions. The research team met to discuss and finalize the initial codes. Any discrepancies between researchers were reviewed together and were resolved after a discussion and a review of the literature. After the generation of the codes, a codebook was developed, and each code was well-defined to ensure no overlap with other codes. These codes were then applied to all transcripts. Following coding of the transcripts, thematic analysis was conducted, and broader themes and subthemes were extracted based on the similarities between codes and the questions asked. Atlas.ti Web facilitated the iterative process of theme construction by allowing codes to be grouped, reorganized, and linked to illustrative quotations. Themes and subthemes were defined and named after undergoing a thorough review to ensure coherence.^27^

## Results

Fourteen pediatricians participated in the interview sessions: 11 general pediatricians and 3 pediatric subspecialists. Each interview was no longer than 45 minutes. All 14 participants had received basic training in screening for ASD using validated tools, and 5 received additional training in diagnosing ASD. Participants had a mean age of 50 years, approximately half were White or Caucasian (50%), a majority were female (79%), worked in a hospital-based setting (64%), and had 3 to 5 years of professional experience (64%; Table 2).

Overall, participating pediatricians reported that they followed recommended national guidelines for screening and diagnosing set forth by the American Academy of Pediatrics (AAP) for all children. Three major themes emerged: (1) barriers causing delays in ASD screening and diagnosis; (2) individualized approaches in pediatric care to address barriers, and (3) recommendations to improve rates of early ASD screening and diagnosis. The themes and subthemes are presented below with example quotes (Table 3).

**Table 3.** Summary of the themes and subthemes with additional example quotes.

Themes and subthemes	Example quotes
1. Barriers causing delays in ASD screening and diagnosis	
1.1. Despite recommended practices, non/late attendance at well-child checks is a major reason for delays in ASD screening	In pediatrics, there’s a lot of things that you’re just observing in the room, or you are understanding more about, like history and parent reports. And honestly, a lot of observation in the room. But we do use the [Modified Checklist for Autism in Toddlers] MCHAT at the 18 month and 24 months checkup. – Participant 2, general pediatrician If the patient is non-compliant for their well checkups on time, they don’t come as recommended then that could be another reason why there could be a delay in the early diagnosis. – Participant 10, general pediatrician So basically, people came late for their well child checks. I would say between April of 2020, in September of 2020, people just didn’t come. And so if your kid turned 18 months in that time, they didn’t get screened. And then they’re turning 2, and maybe they come and they get screened. – Participant 2, general pediatrician There’s this thing that I call it kind of like a sweet spot. You want to identify them early because they tend to do better the earlier, they get the intervention. You know where you can get them enrolled into services and they have this rapidly growing and developing brain and interventions are appropriate for a young brain. So, if we can intervene with them sooner, that’s better for them. – Participant 3, general pediatrician Earlier we screen and diagnose, the earlier we can do interventions. The brain growth and brain development happen at a young age, we are able to make a higher impact if we diagnose earlier and start therapy earlier. – Participant 10, general pediatrician
1.2. Delayed access to specialists due to lower parental understanding of health needs of children with ASD	Parent perception on the diagnosis of autism, and I think there is some stigmatism with it. There are parents who don’t want their kid to be diagnosed with autism or labeled with autism spectrum disorder. And so, sometimes there are barriers on the side of the parents wanting to go with further evaluation. – Participant 6, general pediatrician I think there’s also a health literacy barrier as well, and so not everyone understands what types of questions we’re asking. If we get a lot of questions where, umm, some folks will answer some questions inaccurately and we go back and explain it to them. – Participant 3, general pediatricians Having to translate the M-CHAT for a family that don’t speak English or Spanish could technically be a barrier. I mean, we worked through it by just using communication translation services, but it just takes a lot longer.– Participant 12, general pediatrician I think that [parent] education is really important, and I like that we have these, the awareness months and that we have more number of parents speaking out, parents support groups are very, very important because they help the patient to understand and the parent to understand what are the things that we can do. – Participant 8, general pediatrician
1.3. Lack of general provider clarity on the next steps after ASD is suspected in the child	So, I have another pediatrician that works here, but I don’t think either one of us is comfortable in having the conversation to say your child has autism or directing them. – Participant 4, general pediatrician
1.4. Continued specialist shortages increase accessibility issues delaying diagnosis and therapy initiation	So, if they are Medicaid the only options are to utilize our university-based autism diagnosing practices through our academic center and that wait list can be anywhere up to two years. My experience with ABA and Medicaid is, yes, it was funded [in Texas], but it was underfunded and most of the ABA companies want to take 1% of their clientele as Medicaid. – Participant 3, general pediatrician
1.5. Social determinants of health impact patient access to specialist services	I think that like I mentioned before, there’s a lot of social barriers for children who have no insurance or Medicaid and that have multiple barriers, not just transportation, but there’s financial and housing, you know, all of those. – Participant 13, general pediatrician
2. Individualized approaches to pediatric care to address barriers	
2.1. Pediatrician strategies to help families follow up with specialist referrals	I have often heard parents say, nobody ever called me. I hear that gazillion times and that whether they did get a call from the clinic and left messages, and nobody got the messages, that is probably the most often reason for the parents not getting the appointment. – Participant 13, general pediatrician A few tips I can tell you which I do is when I send the kid to [Early Childhood Intervention] ECI, I also give parents ECI’s phone number. I feel earlier follow-ups rather than waiting for a well checkup helps, and I have seen a good improvement in that. – Participant 11, general pediatrician Now we just have a dedicated person who all day long, is just sitting there and doing referrals, making sure that they have all the right paperwork so that they don’t get rejected or, you know that everything is signed and sent in time. – Participant 8, general pediatrician If [the child] is in school at age 3, we’ll refer them to the school district for an evaluation and see if they can at least start some of the therapies. – Participant 3, general pediatrician
2.2. Resources such as Project ECHO autism model ^22^ helped facilitate ASD diagnosis & shorten wait times during the pandemic	We actually have some of our general pediatricians have gone through additional training to be able to diagnose kids, the younger kids, with their classical autism so that way we can have a little bit of a faster evaluation time frame as well, but the wait time is still a minimum of between like 6 to 8 months for an evaluation. – Participant 6, general pediatrician
2.3. Telehealth supported access and pre-diagnosis data collection but was less effective for direct patient evaluation and interaction.	It’s hard to get a child on the screen. They don’t want to sit, and you can’t really tell anything. Like for kids, you must see them and touch them and interact with them for sickness and for something like a developmental disorder, you have to be observing them. – Participant 2, general pediatrician Unfortunately, doing a medical visit over the screen, doing this all the time and you know the Wi-Fi is terrible and disconnects four or five times and then you have an interpreter that you can’t bring into EPIC or all scripts. You have an interpreter on an iPad who’s talking to a computer screen and to me. So, it was not the most fulfilling situation for anybody. – Participant 7, pediatric subspecialist Setting up sites, where I have the equipment already purchased, give them the toys, the cameras, the laptop and everything and the ADOS kit and then do telehealth where I have control over the camera in the room and I can follow the kid around and see what he’s doing, and there is a facilitator in the room like a medical assistant who could help facilitate the visit would be beneficial. – Participant 7, pediatric subspecialist
3. Recommendations to improve rates of early ASD screening and diagnosis	
3.1. Additional ASD-related training for general providers to improve patient care	I am always up for like education. I feel like there’s still [uncertainty], me and my colleagues are talking between ourselves like you know, does anybody have this kid? I don’t even know the best place for them or what do they need. – Participant 1, general pediatrician So, if there was something like once every few months, some type of CME or update, you know like is there more that we could do to be updated. Is there, you know, anything else that we can do versus just being kind of the middleman that would be awesome. – Participant 1, general pediatrician
3.2. Need for clinic support systems improve parent confidence in advocating for their child’s needs	What helps facilitate it is having support from our social workers as far as access, like having somebody to help you advocate for your child, because not all parents can navigate the school system really well and making sure you're advocating if your child needs like an individualized education plan or an [Individualized service plans] ISP or, you know, accommodations for ASD. And I think having those people to help provide support. – Participant 6, general pediatrician

*Theme 1: Barriers causing delays in ASD screening and diagnosis*
*Subtheme 1.1: Despite recommended practices, non/late attendance at well-child checks is a major reason for delays in ASD screening*

There was a common consensus among general pediatricians that screening delays occur due to children not attending routine well-child checks at 18 and 24 months of age. General pediatricians working with majority Medicaid recipients reported greater issues with non-attendance with well-child checks. The COVID-19 pandemic further exacerbated this issue and resulted in a drastic decrease in the number of children attending well-child checks. Clinics are still trying to catch up on all the missed visits and screenings.

* “We have a dramatic drop off in the number of children that came in for well-child visits [during the COVID pandemic]. All those kids also had a significant drop off in screening for autism and other conditions as well. So, I think we are still doing catch up with those.”* – Participant 9, general pediatrician

Overall, general pediatricians reported following recommendations related to screening for children who came in for routine well-child checks. They emphasized the importance of screening for ASD at the recommended screening age at 18 and 24 months on child development; however late or non-attendance at well-child visits contributed to the delays in screening. There was agreement across pediatricians that current ASD screening tools must be accompanied by information from interactions with the child and parent-provided feedback.

*Subtheme 1.2: Delayed follow-up to specialists due to lower parental understanding of health needs of children with ASD*

Participants indicated that parents may struggle with accepting their child might have ASD during initial screening and subsequently delay an evaluation to receive a formal diagnosis. Pediatricians reported that parental hesitancy often stems from the parents’ perceptions of ASD, prior experiences with ASD, and their general health literacy. Therefore, many parents choose to “wait and watch” before seeking additional care for ASD. Participants reported some parents not understanding the questions included on the ASD screener, resulting in inaccurate reporting of their child’s behaviors. Parental comprehension issues also arise if the screening tools are not available in a language the parent understands.

* “A lot of parents are very hesitant to admit that their child might have that sort of diagnosis and so I find that there are a lot of parents who just kind of want to wait and see.”* – Participant 4, general pediatrician

Due to a potential lack of understanding from parents and parental difficulties in accepting their child might have ASD, participants emphasized the importance of appropriate and sensitive provider communication with the parent coupled with parental education. However, some general pediatricians with less experience with ASD did not feel confident with having these discussions.

*Subtheme 1.3: Lack of general provider clarity on the next steps after ASD is suspected in the child*

Subspecialists reported that while many general pediatricians are providing the screening across the country, not all are equipped with sufficient understanding of the next steps after a positive screen and the best practices. In particular, subspecialists reported that they have observed general pediatricians encouraging parents to wait and watch instead of directing them to specialists.

*“Unfortunately, there’s a lot of providers in the general pediatric setting who are continuing to encourage parents to watch and wait and to you know let kids kind of develop naturally and just see how things are and not get very nitpicky on their development and then some have already mistakenly said no they’re fine or they don’t have a diagnosis now and it’s parents who are pushing for it and that delays things as well.” –* Participant 5, pediatric subspecialists

*Subtheme 1.4: Continued specialist shortages increase accessibility issues delaying diagnosis and therapy initiation*

All pediatricians stated that shortages of specialists trained to provide an ASD diagnosis has continued to be a significant issue, resulting in longer waiting times for diagnosis and therapy initiation.

*“There hasn’t necessarily been a lot more people doing ABA or a lot more people doing diagnosis. We still struggle with the whole issue of lack of providers in regions, especially providers that take Medicaid.* – Participant 2, general pediatrician

Post-screening, many children wait between 6 and 12 months for a diagnosis and an additional 6 months to receive key therapies such as ABA or speech therapy. Medicaid recipients tend to have even longer wait times. Unfortunately, not all specialists take Medicaid insurance, leaving Medicaid recipients with fewer options for ASD diagnosis and therapies and longer wait times.

*Subtheme 1.5: Social determinants of health impact patient access to specialist services*

There was consensus among participants that many low-income and Medicaid recipients tend to have additional external factors that impact their access to services, such as transportation issues and financial strain.

*“You know there can be some variabilities in, like, transportation to and from services, parents getting time off work to go to these services.”* – Participant 6, general pediatrician

These external factors may affect their access to healthcare and potentially delay access to key services and therapies.

*Theme 2: Individualized approaches in pediatric care to address barriers*
*Subtheme 2.1: Pediatrician strategies to help families follow up with specialist referrals*

Practices after a positive screening tended to depend upon pediatrician experience and knowledge, along with parent engagement. Several general pediatricians in this study reported placing multiple referrals to specialists for further assessment or therapies after a positive screening. However, at the next visit, typically a year or so later, parents were reporting not being contacted by the specialist, thus not being able to access necessary services for their child. It was noted that lower socioeconomic populations, including those on Medicaid, might need more guidance navigating the different resources than others.

*“Sometimes our lower socioeconomic groups kind of need a little bit of hand holding understanding the different referrals.” –* Participant 1, general pediatrician

Several general pediatricians incorporated better follow-up practices to ensure families were able to access these services. Practices included following up with patients after the appointment to see if a specialist appointment was scheduled, providing parents with the specialist’s contact information and encouraging them to reach out, and leveraging or hiring clinic staff to help follow up with patients. Participants also mentioned referring school-going patients to school-based specialists to facilitate a diagnosis and therapy initiation to prevent further delays.

*Theme 2.2: Resources such as Project ECHO autism model* ^28^ *helped facilitate ASD diagnosis & shorten wait times during the pandemic*

During the COVID-19 pandemic, to improve access to diagnostic services, 2 general pediatricians practicing in a hospital setting reported getting trained in Extension for Community Health Outcomes (ECHO) autism model that uses the Screening Tool for Autism in Toddlers and Young Children (STAT), an ASD diagnostic tool, to help facilitate diagnosis for young children and improve accessibility across the region.^28^ This model has also been successful at reaching rural providers and psychologists and decreasing wait times.

*“There’s a model called the ECHO model. They’re doing a really good job. The original one is out of University of Missouri and basically trains our general pediatricians in learning how to do STAT. I’m happy it’s helping to reach out to rural providers and psychologists.” –* Participant 4, general pediatrician

*Theme 2.3: Telehealth supported access and pre-diagnosis data collection but was less effective for direct patient evaluation and interaction*

During the initial phase of the COVID-19 pandemic, telehealth was used in some participant clinics to address the decline in well-child checks. However, general pediatricians stated that telehealth was not as successful as in-person visits because interaction with the child was not possible.

* “It’s hard to get a child on the screen. They don’t want to sit, and you can’t really tell anything. Like for kids, you must see them and touch them and interact with them for sickness and for something like a developmental disorder, you have to be observing them.”* – Participant 2, general pediatrician

Subspecialists also incorporated telehealth into their practice to diagnose children with ASD. Telehealth helped facilitate the initial patient intake and history portion of the appointment but was less successful with the actual evaluation due to technology issues.

* “During that time, we use telemedicine and, in some cases, especially for obtaining like history and developmental histories and other evaluations on symptoms.”* – Participant 14, pediatric subspecialist

However, the subspecialists did agree that developing structured telehealth sessions for ASD evaluation, with the proper infrastructure, could help with access issues among rural communities and those with transportation issues.

*Theme 3: Recommendations to improve rates of timely ASD screening and diagnosis*
*Subtheme 3.1: Additional ASD-related training for general providers to improve patient care*

General pediatricians were open to additional education and training if it would help improve provider-patient communication regarding ASD and facilitate earlier diagnosis. Many were not aware of any trainings that are currently available.

* “And that would be like one thing I know sometimes they just like kind of educational talks, but then too that can be helpful for [general] pediatricians and other people that are screening to kind of like give them this viewpoint [and] advice.*” – Participant 14, pediatric subspecialist

Subspecialists also suggested that improving collaboration between general pediatricians and subspecialists could help in improving general pediatricians’ understanding of ASD and help streamline referrals to diagnostic services. Particularly, sharing experiences and knowledge with general pediatricians can help provide more guidance.

*Subtheme 3.2: Need for clinic support systems to improve parent confidence in advocating for their child’s needs*

Pediatricians recognized that ASD is an extremely complex condition, resulting in hesitation from parents to get a diagnosis for their child and results in a need for additional support.

* “So having a social worker on site is also very helpful because what if this parent goes home and their phone is not reachable even if the social worker tries to contact them.” –* Participant 11, general pediatrician

They emphasized the need for greater clinical support/care coordination in the form of community health workers, social workers, and referral specialists, to ensure parents fully understand what an ASD diagnosis means for their child and to help them advocate effectively for their child with schools and other specialists.

## Discussion

To our knowledge this study is one of the first to provide a qualitative investigation as a systematic approach to understanding the nuances of barriers and strategies to ASD screening and diagnosing. General pediatricians’ and pediatric subspecialists shared insights on barriers and strategies, drawing on their experiences with underserved populations. Pediatricians in this study continued to highlight significant barriers that have been identified in past literature^29-36^; this study adds context to those findings in the literature by providing specific insights from the perspective of pediatricians who serve underserved populations. Low patient attendance to routine well-child checks continues to be a significant barrier reported by participants, particularly among pediatricians seeing large proportions of Medicaid recipients, thus further emphasizing the need to continue the work with parents to understand and address the barriers resulting in non-attendance.^29-31^ Engaging the health system leaders and clinic staff through multi-disciplinary collaborations is important to ensure barriers are met.^37^ Multidisciplinary collaborations that include community health workers or social workers who work with general pediatricians and pediatric subspecialists to assist families in navigating social services and the healthcare system can improve attendance at well-child checks and subspecialty visits.^38^ Past studies have shown increases in well-child checks and improvements in continuity of care after the incorporation of telehealth, along with greater use of tools and resources to help address barriers faced by patients and combining different appointments to reduce the number of visits to hospitals or clinics.^37,39^

Pediatricians in our study reported that despite overall improvements in awareness of ASD among the public, parental hesitancy persists, especially among underserved populations.^40^ This finding closely aligns with past findings that parental understanding of ASD impacts whether their child will see a specialist.^32^ Cultural norms, ASD-associated stigma, and low parental education play a role in continued hesitation.^40^ The pediatricians included in the current study also described similar issues in their practice. While additional training has been developed to improve evaluating ASD in primary care,^18,19^ this finding highlights the importance of ensuring pediatrician training also focuses on cultural sensitivity and implicit bias to ensure empathetic and engaging communication between the pediatrician and parent, in addition to continued awareness and education campaigns targeting parents. The suggestion by pediatricians in this study for parental support is aligned with the existing literature - parental support groups facilitated by primary care clinics or communities can also help improve parental awareness.^33-35^ In addition, it is necessary to conduct qualitative work with parents to gain insight into their lived experiences regarding screening and diagnosing for autism among children when developing healthcare strategies to improve ASD screening and diagnosis follow-up.

Participants reported different pediatrician practices following a positive screen-increasing general pediatricians’ capacity and confidence in addressing ASD concerns with families should be a priority as.^18,19,32^ Trainings targeting general pediatricians’ knowledge and confidence on ASD are available,^18,19^ but are not part of routine required medical education. In fact, only 3 of the 11 general pediatricians in this study received any additional ASD-specific training aimed at improving their confidence and knowledge on communicating with parents. This highlights the need for stronger evidence detailing the benefits of incorporating these trainings into systematic, routine education for general pediatricians. Additionally, our participants indicated that the lack of patient access to specialists who can formally diagnose and provide therapies for children continues to be a significant barrier to an early ASD diagnosis.^12^ Studies have shown that improvements in parental and general pediatrician awareness, identification, and understanding of how ASD manifests in children have resulted in greater demands for subspecialists who can diagnose ASD, which raises national concerns regarding whether the pediatric workforce can sustain the demand.^41,42^ These demands have resulted in declines in medical school graduates applying for pediatric residency programs and decreasing interest in subspecialties.^43^ This has resulted in many pediatric subspecialty positions remaining unfilled,^41^ emphasizing the need for improving general pediatricians’ knowledge by potentially leveraging the use of validated models, such as ECHO Autism, and increasing efforts focused on promoting collaboration between general pediatricians and subspecialists.^28,44^

In addition to the shortage in the pediatric workforce, the process for accessing specialists can be complicated after a referral from a pediatrician. The continued lack of health system-wide, effective care coordination makes it difficult for families to navigate specialist referrals, particularly those reporting additional unmet non-medical needs.^45^ There also seems to be a need for increased care coordination and communication between general pediatricians and pediatric subspecialists as there were notable differences in perceptions regarding follow-up after a positive screen. General pediatricians reported that parents typically wait for a diagnosis, while subspecialists indicated that general pediatricians often advise parents to wait. Therefore, standard practices should be developed to help streamline the referral process for specialist services. These practices could include having a referral manager to help parents navigate the referrals and following up with the patient and parent a few weeks after the initial appointment to determine what additional assistance is needed.^46^

An added layer brought forward by the participants of this study was the limited number of specialists who accept Medicaid, thus increasing demand and resulting in longer wait times for diagnosis and therapies for Medicaid recipients. Medicaid reimbursement rates are low, which results in fewer providers willing to accept Medicaid – it adds additional burdens on the provider without adequate compensation.^47^ Thus, children on Medicaid are limited to a smaller pool of providers, resulting in fewer appointment availabilities.^47^ This may exacerbate disparities associated with the timing of when children receive an ASD diagnosis and subsequent therapies. Therefore, policy considerations such as improving reimbursement rates for Medicaid recipients should be a priority to increase access to services and decrease disparities.^41,48^

This study also highlighted the importance of screening for social determinants of health (SDOH) and understanding patient demographic characteristics, especially in clinics serving low-income, uninsured, and Medicaid recipients. SDOHs are external non-medical circumstances that impact a person’s living, working, and social environment and have significant impacts on their health.^49^ These factors include transportation issues, access to care, health literacy, and financial strain. These determinants were reported by pediatricians in this study and previous studies as potential reasons for delays in ASD screening and diagnosing.^50^ Participants highlighted that unmet SDOH needs among families with a child suspected of having ASD adds additional strains to the household and thus might explain the missing of appointments with specialists. Therefore, screening and addressing SDOH concerns has been recognized as an imperative part of general patient care.^51^ However, universal SDOH screening in pediatric primary care settings is yet to be established.^51^ Universal SDOH screening and follow-up are essential to help families overcome barriers to care. Additionally, understanding differences in screening and diagnosing based on sociodemographic characteristics, such as socioeconomic status, and SDOH is important to allow for an equity-centered approach. Approaching ASD screening and diagnosing delays from a health equity lens allows for the appropriate tailoring of solutions based on patient and family experiences.^52^

Lastly, this study highlighted that impacts of the pandemic are still prevalent. Post-pandemic work is needed to ensure no child foregoes vital screenings and or services necessary for growth and development.^53^ This study also adds a novel perspective on the use of telehealth for ASD diagnosis. Subspecialists reported that while telehealth facilitated pre-diagnosis information gathering, it was less effective in supporting the diagnostic process itself due to the lack of appropriate infrastructure. Therefore, for it to truly be successful and incorporated into daily practice, the infrastructure must be built to ensure families can access the services. Similar to past studies, our study found that telehealth helped to expand the geographic reach of ASD diagnostic services and improved access to ASD care.^54^ Therefore, healthcare facilities should explore ways to improve and expand telehealth services, to reduce delays in ASD diagnosis.

### Limitations

This study helps bridge the gaps in the current understanding of delays in ASD screening and diagnosing, by providing insights from general pediatricians and pediatric subspecialists who serve underserved populations. A review of the qualitative studies related to ASD highlighted the impact of qualitative research in providing more concrete solutions when addressing the disparities associated with ASD.^11^ There are limited qualitative studies aiming to understand the delays in ASD screening and diagnosis; therefore, this study helps close that gap and provides insights into concrete solutions to address potential barriers. However, this study is not without limitations. Firstly, this study was limited to pediatricians in the Greater Houston area in Texas, thus limiting the number of pediatric subspecialists meeting the eligibility criteria for study and may limit the generalizability of our study’s findings since the experiences of pediatricians in other states might differ from those in Texas. However, most pediatricians interviewed as part of this study have practiced in other states and stated similar experiences across states. Secondly, since this study followed a qualitative design, we were unable to quantify the extent of the impact of identified barriers and facilitators. However, we did recruit pediatricians from different institutions and across the city to ensure the inclusion of experiences in different patient populations. Additionally, further research needs to be conducted among parents to get a holistic understanding of the barriers in timely ASD screening and diagnosing and ensure the development of beneficial practices and strategies.

## Conclusion

This study highlights pediatricians’ perspectives on the factors preventing children from receiving an ASD diagnosis early. While this study also highlights individual strategies and trainings utilized by pediatricians to overcome these factors, there continues to be a lack of health system wide practices resulting in ongoing persistent barriers. There continues to be significant shortages of specialists, lower general pediatrician confidence in addressing ASD, parental hesitancy, and lack of health system-wide care coordination. Participants did highlight that underserved populations tend to face more barriers. Medicaid recipients have greater access issues because not all specialists accept Medicaid. There is also a lack of system-wide care coordination after a positive screen which can add additional burdens for families who are also experiencing unmet SDOH needs. This study also provided new perspectives on the use of telehealth in screening and diagnosing. Going forward, health systems should work to expand and evaluate identified strategies using a health equity approach to ensure strategies are tailored based on population needs.
